# Contemporary Artists’ Books and the Intimate Aesthetics of Illness

**DOI:** 10.1007/s10912-019-09596-4

**Published:** 2019-12-27

**Authors:** Stella Bolaki

**Affiliations:** University of Kent, Canterbury, CT2 7NX UK

**Keywords:** Illness narrative, Artist’s book, Intimate authority, Temporality of illness, Mindfulness

## Abstract

This essay brings together critical perspectives from the discrete traditions of artists’ books and the medical humanities to examine artists’ books by three contemporary artists – Penny Alexander, Martha A. Hall and Amanda Watson-Will – that treat experiences of illness and wellbeing. Through its focus on a multimodal and multisensory art form that has allegiances with, but is not reduced to, narrative, the essay adds to recent calls to rethink key assumptions of illness narrative study and to challenge utilitarian approaches. In particular, it draws attention to the aesthetic and imaginative elements of illness communication by exploring how artists’ books represent lived experiences in a distinctively palpable way and offer an “intimate authority” that extends beyond narrative legitimacy or a form of struggle against the medical gaze. By interrogating narrative’s dominance in medical humanities research, the essay further expands awareness of illness experiences that resist conventional forms of representation (such as chronic illness), and of alternative reflective practices within healthcare education that encourage engagement with both mind and body.

This essay brings together critical perspectives from the discrete traditions of artists’ books and the medical humanities to examine artists’ books by contemporary female artists who treat lived experiences of illness and wellbeing. Through its focus on a multimodal and multisensory art form, it adds to recent calls to rethink scholarly and cultural approaches, as well as professional practices, that favor typologies and certain qualities of illness narratives or reduce them to medical data. In particular, it draws attention to the aesthetic and imaginative elements of illness communication: it examines the distinctive strategies through which artists’ books mediate embodied experiences and offer an “intimate authority” (Drucker 2011, 14) that extends beyond narrative legitimacy or a form of struggle against the medical gaze. By interrogating narrative’s dominance in medical humanities research, the essay further expands awareness of illness experiences that resist conventional forms of representation, and of alternative reflective practices within healthcare education that encourage engagement with both mind and body.

Clinicians, medical humanities scholars, and members of the public have privileged particular ways of presenting or sharing knowledge about health such as third person reports, medical data, and representations of illness experience that rely on language or emphasize linearity, coherence, and closure. This phenomenon not only generates “epistemic injustice” (Kidd and Carel 2017) but also ignores the “aesthetic achievements” of illness narratives (Willis et al. 2013, 63): for example, their historical and intertextual connections that situate them in relation to various “imaginative communities” rather than exclusively within the context of biomedicine (Ibid, 65). Many scholars who affiliate themselves with the second ‘wave’ of the medical humanities have criticized utilitarian approaches that “unnecessarily restrict what illness narratives might be allowed to mean, and even what they might look like” (Waddington and Willis 2013, iv). They have argued in favor of engaging “with an expanded notion of literary genre,” including more experimental and “mixed-media narrative modes” that redress the dominance of realist fiction and autobiography in existing scholarship (Whitehead 2014, 114). According to Angela Woods (2011), the field should not only “foster a critical approach to the normative scripts of particular *kinds* of narrative” that promote an authentic and private self, but it should also “more radically” move beyond narrative by embracing fragments, metaphors, and art forms such as photography and performance art that resist “closure and containment in the pursuit of the paradoxical, the ambiguous and undecidable” (76). In addition to exploring the value of textual fragments, episodes and moments, Sara Wasson has since made an intervention into these debates by showing how affect theory can expand the existing critical vocabulary in the more specific context of representations of chronic pain. In an essay aptly titled “Before narrative,” she writes that “rather than foregrounding the coherence of some illness representations, we can recognise how value may inhere in the rupture and the breach” and calls for the need to “heed these traces of embodied suffering before they solidify into story” (Wasson 2018, 6).

As it does not relinquish its allegiances to either art or to the book, the artist’s book is a productive format for rethinking debates about narrativity and the role of affect in representations of illness experience. Despite the inherent association of book structure with sequence (the most common book form is the codex, which is made with pages fixed in a rigid sequence by being clasped on one side), sequence in artists’ books can operate within narrative and non-narrative frameworks. Moreover, if we consider their materiality, artists’ books occupy an intermediate ground between embodied or affective experience and narrative structure. The affective dimension of artists’ books is evident in the kind of reading they encourage. Readers must learn to ‘perform’ them as much as ‘read’ them. Handling artists’ books requires not only interacting with words and images but also paying attention to their shape, size, format, color, texture, typography and even fragrance and sound. Their advantage over literary narratives of illness is that they invite a participatory touch that makes them interactive, sensuous, and intimate forms of exchange. As Johanna Drucker (2004) writes, “We enter the space of the book … we are in a physical relation to the book. The scale of the opening stretches to embrace us, sometimes expanding beyond the comfortable parameters of our field of vision, or at the other extreme narrows our focus to a minute point of intimate enquiry” (360).

While its intimacy partly explains the ongoing appeal of the artist’s book for some artists, in this essay I want to explore the value and contribution of this medium’s distinct aesthetic qualities to illness narrative study by specifically reading artists’ books that address illness. As a vehicle of both private expression and broad communication, the book balances “enclosure and exposure” and offers “intimate authority” (Drucker 2011, 14). The negotiation of enclosure and exposure may resonate with the lived experiences of many female artists, but it also has specific applications to lived experiences of illness. Given the stigma and emotional difficulties surrounding conditions such as mental illness, for example, some people may find the ‘private-public’ nature of books a more suitable vehicle of communication than other art forms that have a more public presence. Unlike paintings, sculptures and films, artists’ books are created for one-to-one interactions (this does not mean that they cannot reach many people). A balance between enclosure and exposure is not only relevant for the maker of a book but also for their audiences who, in the case of work exploring illness, are often invited to witness pain and suffering. The intimate ‘embrace’ of the artist’s book may be perceived as threatening; as Martha A. Hall (2003), one of the artists I discuss later, acknowledges: “People may not want to ‘touch’ the topics I explore in my books; yet the books invite handling, touching, interaction” (14).

Reading artists’ books that treat illness experience as “works of illness” (Radley 2009) can help broaden the meanings of “intimate authority” beyond a strict emphasis on the art world. But, as I further argue in this essay, building on Martin Willis, Keir Waddington and Richard Marsden’s approach (2013), reading illness stories by “investing” in a different kind of “community,” such as the tradition of artists’ books, can provide an additional form of authority for the patient/artist, which is as important as reclaiming one’s voice from the depersonalizing discourse of medicine via narrative (68). If medicine is associated with a particular kind of authority and expertise to which the patient often needs to surrender, book artists, like writers of illness narratives, claim a different kind of authority in articulating lived experiences that are not to be found in x-rays, laboratory studies and pathology reports through their work. The fact that artists’ books “literally process” illness experiences, “an introspective exercise made visible through stitches, glue and tears” (Wellbery 2017, 19), is integral to the alternative knowledge – corporeal, emotional and intimate – that this art form and an “aesthetic epistemology” more broadly (Willis et al. 2013, 63) offer to the representation of illness experience.

In light of their multimodality, the intimate authority of artists’ books cannot therefore merely be subsumed within the restorative power of storytelling that has been covered substantially in the illness narrative field (see for example Frank 1995). It is also important to consider the specific kinds of agency or affective sustenance associated with making (including with particular bookmaking strategies and techniques) as well as with ordinary forms of creativity beyond narrative that take us past the traditional boundaries of the medical encounter. Audrey Niffeneger (2011) writes that to make a book means to “gain power over objects”; holding her first book in her hands, suddenly she realized she “had made *a thing*,” which felt radically different from what she had previously made, namely “images and text” (13). Similarly, Drucker (2004) alludes to the “air of authority” conferred by the acts of printing and binding (xviii). While binding gives fragments coherence –“otherwise the disorder would be meaningless, unbounded” (Ibid, 127) – printing “provides a fundamental means of transforming personal expression into an authoritative form within the social order and the public sphere. The physicality of printing makes that transformation a somatic experience, an act of the body, which moves the interior voice, the personal word, into the cultural domain” (Ibid 1998, 4). Therefore, for a patient/artist who makes a book about her illness experience, her authority does not derive merely from reclaiming her lived experience by refusing to surrender to medicine’s grand narrative. It also comes from putting her work into dialogue with other artists’ books, techniques, and aesthetic traditions that allow it to “accrue meaning” beyond the clinical gaze (Willis et al. 2013, 65).

Woods (2011) has warned that narrative “promotes a specific model of the self as an agentic, authentic, autonomous storyteller; …as someone who possesses a drive for storytelling, and whose stories reflect and (re)affirm a sense of enduring, individual identity” (74). This has implications for anyone who for various reasons is unable or unwilling to communicate her illness experience in this way. While artists’ books can promote a similar model of self, they don’t have to. Eve Kosofsky Sedgwick (2011) has written about how the making of her textile books to which she turned in order to explore her mortality (when her breast cancer had become incurable) did not involve “the construction of an identity, nor a change of identity, nor even the deconstruction of one, but something very different: a meditative practice of possibilities of emptiness and even of nonbeing” (69). In “Making Things, Practicing Emptiness,” Sedgwick associates speech, writing, and conceptual thought with “the fantasy of omnipotence” whereas “working with physical materials – paper, fabric, thread and other supplies press[ing] back so reliably, so palpably against my efforts to shape them according to models I’ve conceived” offers “the reassuring sense of a grounding in reality.” This is because the “will of the artist” is “only one determinant” of the art that emerges; the final outcome involves a negotiation between what the artist wants to make and what the materials will let her make. Sedgwick concludes: “It feels wonderful to exist and to be active in that space of suspended agency” (Ibid, 83). This form of agency is not antithetical to the experience of intimate authority, but it is not predicated on the idea of mastery or cohesion of the self.

In the following sections I turn to three artists who have explored lived experiences of physical and mental illness and wellbeing through the unique versatility and communicative power of the artist’s book medium: Penny Alexander, Martha Hall, and Amanda Watson-Will. While adopting different formats and structures, *Mind Maps, The Rest of My Life II*, and *Like Weather*, which featured in the exhibition *Prescriptions: Artists’ Books on Wellbeing and Medicine* (Beaney Art Museum, Canterbury, April–September 2016), highlight the book format’s close relationship to time. In turn, this link between the book and time opens up for examination some of the aforementioned questions about the contested uses of narrative in the medical humanities field. Time is medicine’s central axis not only in terms of the process of diagnosis, treatment and cure, through which disease is understood, but also the “temporal experience of illness at the subjective level of the patient” (Toombs 1990, 227). As the subsequent analysis demonstrates, artists’ books can provide invaluable insights into a range of embodied experiences, offering in the process an intimate authority that encourages the medical and health humanities community to rethink key assumptions of illness narrative study and of healthcare education.

## Intimate maps and dotted life-lines

Book artist Rosie Sherwood (2017) asks: “What is a map? What does it do? …Can a map be a reflection of a place, an experience, an emotional response? …Can a map show a moment in time? (75) All the above are true in the case of Penny Alexander’s *Mind Maps* (2016) that charts the relationship between environment and emotional experience to illustrate different states of mind. Subtitled, *The* C*umulative (Hidden) Experiences of the Life of Penny Alexander*, it is a white codex book that was made as a form of “creative recovery” from post-natal depression. Produced with a 1970s German typewriter on watercolor paper, it documents transitional moments in the artist’s life through a series of maps and lists. The book’s ‘scientific,’ conceptual look doesn’t reveal its connection to illness or emotional experiences at first glance. The left side of each spread contains a map formed by lines of black full dots. There is nothing else on the maps other than the dotted lines and an X mark to indicate Alexander’s home address. The right side provides a ‘key’ to each map containing the location (in the form of a postcode rather than a town’s name) and the dates the artist lived in that location (covering in chronological order the years 1986–2016), as well as a list of experiences viewed as “telling indicators of a person’s overall contentedness” (Alexander 2017, 5). Every experience on the list comes with a number next to it that indicates frequency of occurrence – a way of quantifying the well-being of living in a particular place. In Alexander’s words (2018), “a brief outline of experiences and a tally system seemed like a powerfully impassive way to express myself whilst still maintaining honesty and some sort of privacy.” To recall Drucker’s terms, Alexander balances enclosure and exposure through the book format and through the particular structure adopted in this book.

**Fig. 1:**
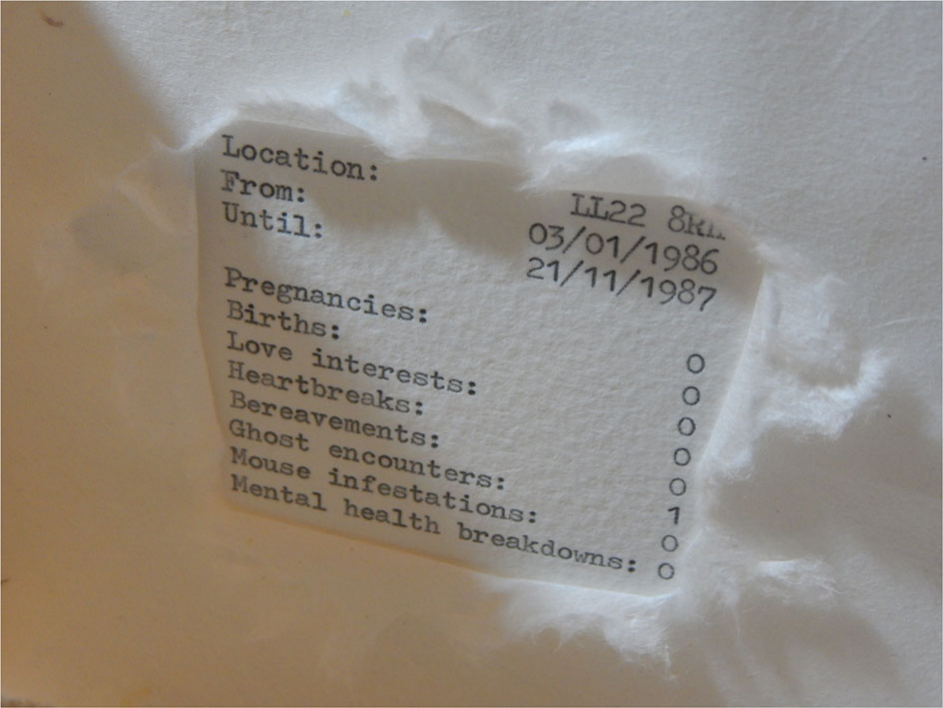
Penny Alexander, *Mind Maps* (2016). Typewriter on watercolor paper with Japanese paper overlay. Special Collections and Archives, University of Kent. Page from the book displaying outline of experiences and tally system

Instead of an elaborate narrative, with *Mind Maps* Alexander (2018) offers readers clues and links along the way “to uncover, relate to and hopefully feel a sense of kinship with the author.” Even though it wasn’t in her “nature to shout” about her mental health, as she explains about her process, the book needed to include “a *setting free* of [her] secret. It needed to be quick, like removing a plaster or removing a bandage.” Alexander opted for “mental health breakdowns” for this category, presented last on her list, “matched in weight” with “pregnancies,” “births” “bereavements” and “heartbreaks.” To balance out these deeply personal experiences, she also included more mundane, everyday and relatable ones such as “love interests” and “mouse infestations” (Alexander 2018). There is also a more ambiguous category – “ghost encounters” – which conveys moments of being “haunted,” whether these refer to unsettling emotional experiences or supernatural activities (it may also explore the connection between the two, in the sense that one’s psychological vulnerability and loss of control might be given a “palpable explanation in the form of ghosts”). White semi-translucent Japanese paper partly obscures some elements, turning the lists into semi-secret hiding places that further interrogate the boundary between the private and the public in this unconventional episodic diary. With its soft texture, the paper creates an interesting tension between the organic and the mechanical aspects of the book’s production (Fig. 1).

The maps on the left hand side of each spread are accurate (as much as a map can be accurate) when compared to more conventional cartographic maps, and the postcode given in the ‘key’ adds to the fact-based approach of the book. Ultimately, however, as a series of mental landscapes *Mind Maps* documents how the artist sees and remembers each place, and how moments in time associated with different locations accumulate to chart a life or a particular part of one’s life as in a diary or memoir. Though lacking detail such as street names, the maps offer visually subtle clues about the artist’s emotional experiences which become more meaningful when read alongside the written clues in the tallies they are partnered with. As Alexander (2018) explains:

Looking at the map, the viewer would be able to study the complexity and forms of streets, as well as the quantity of streets. This would be another clue: densely populated urban areas have frequent instances of crime, as well as vermin issues ….Sexual assaults are also commonplace, and as a recipient of some of those, it was also a strong hint at the state of my well-being. I wasn’t lonely in the city, and mouse infestations didn’t lead to mental health breakdowns, nor did sexual assaults, but those environmental factors aren’t deductive of a particularly contented life.

**Fig. 2:**
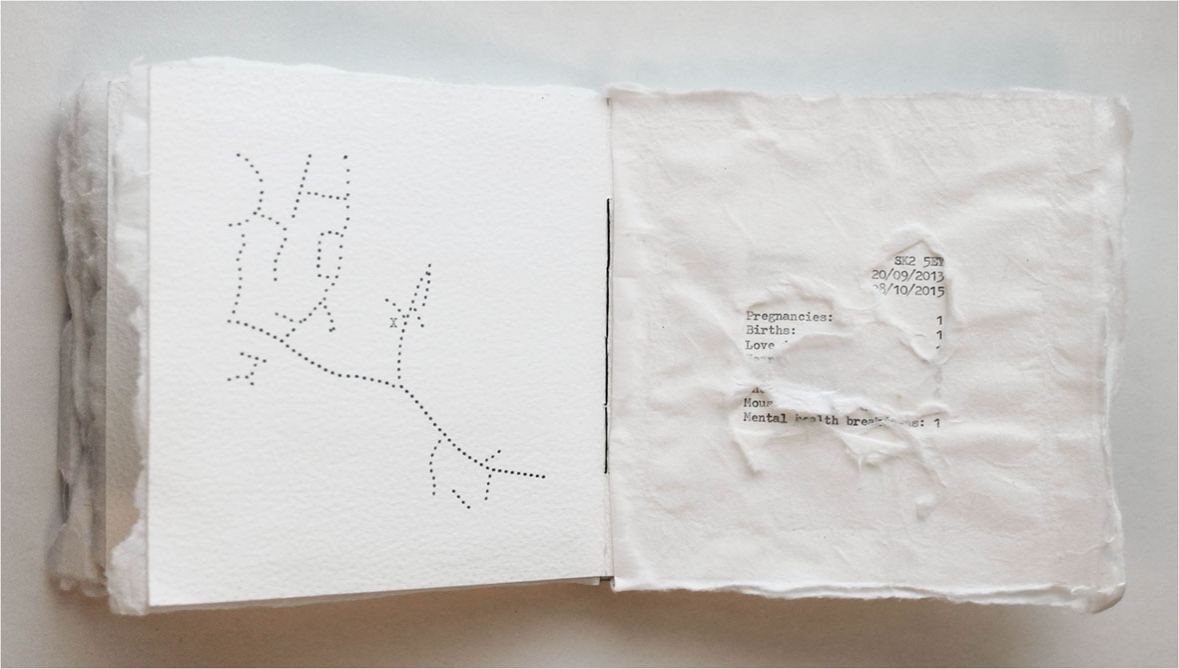
Penny Alexander, *Mind Maps* (2016). Typewriter on watercolor paper with Japanese paper overlay. Special Collections and Archives, University of Kent. Page displaying one of the book’s maps and its ‘key.’ Photo by Egidija Čiricaitė

Studying one of the maps and its corresponding tally (this refers to the period from 20/9/2013 to 28/10/2015), readers piece together the clues offered to form a picture of Alexander’s state of mind. The map has few streets, indicative of the time she lived in the woods with no company or sufficient daylight. The experiences from the list that are not obscured in the ‘key’ part of the spread, showing a clear correlation to each other, are: Pregnancies: 1; Births: 1; Mental Health breakdowns: 1. This is the only page where the category “mental health breakdowns” appears with the number 1 next to it (in all other instances, it is a 0). The meandering lines of the maps may seem cryptic, but as Alexander (2018) is right to note, “Choose to look and it’s all there. Have I ever had an abortion? Have I ever been mugged, burgled, raped? How is my mental health? It’s an open book.” (Fig. 2)

So far I have examined the ways in which *Mind Maps* structures a relation between the private and the public (or enclosure and exposure) in order to communicate mental illness. I now want to turn to the different responses that Alexander’s maps, made exclusively of dotted lines, may elicit in viewers. In his interdisciplinary study *Lines*, anthropologist Tim Ingold argues that the line in the course of its history has been gradually deprived of the movement that gave rise to it, just as the materiality of writing as inscription has been forgotten as a result of print. Lines in modernity become fragmented into a succession of points or dots. The dotted line is a perfect embodiment of that for Ingold (2016): “take a line described by movement, cut it up into segments, roll each segment tightly into a dot, and finally join the dots” (114). Thus, places that were once kinds of knots tied from multiple and interlaced strands of movement have now been reduced to the status of nodes in a static network of “connectors.” Modern maps, like texts, appear as pre-existing plans, tracing a movement from A to B without the movement between these places mattering any more: “They enable the prospective traveller to assemble a route-plan, in the form of a chain of connections, and thereby *virtually* to reach his destination even before setting out” (Ibid, 88).

As ‘mind maps,’ Alexander’s maps are not conventional, as already noted. Moreover, their elliptical nature becomes a powerful metaphor for mental illness. Street names are not included because they “were irrelevant … I was still struggling significantly with my mental health and those pavements I had pounded as a free and sound-of mind person seemed like long *lost roads*” (Alexander 2018, emphasis added). With regards to the dots her map lines are cut up into, Alexander has explained that on returning to her typewriter art, initially a lack of confidence limited her into only utilizing a single symbol; the ellipsis: “It made perfect sense. At that point I didn’t perceive a future (cue ‘…’) but I needed an end to the mental turmoil and to feel ok again on a long term basis (cue ‘.’) Thus meandering lines of ellipses and full-stops were born, with no tangible beginning or end. Much like a metaphor for mental illness” (Ibid).

If according to Sherwood (2017) book artists are able to “un-flatten” conventional maps in all sorts of ways (75), can they also restore their lost movement? Ingold (2016) uses the idea of the “surveyor’s walk” to describe the construction of a cartographic map: surveying is merely a process of collecting data without playing any part “in the integration of the information obtained”; a mode of “occupation” not “habitation” (91–2). Through the dotted lines of her maps, Alexander may not be “enacting” movement on actual geographical ground (Ingold 2016, 88), but she is still engaged in a form of traveling that draws meaning from her lived experience. Borrowing Wasson’s (2018) eloquent phrase, her maps bear witness to the “lines of force” that shape the “affective experience” of the moments she is sharing through the book (4). When we consider the physicality of typing, “force”– and “labour,” another word Wasson draws attention to in her analysis of chronic pain representations (Ibid, 3) – is conveyed quite literally as seen in Alexander’s description of her creative and therapeutic process, which is worth quoting at length:

The ellipsis stood for more than a convenient conceptual and contextual grammatical tool. As I hammered that key I felt like I was using my hands and my mind to pace those streets. I would fall into a trance, in a state of such calm, that one symbol became footsteps, memories and emotional obstacles. Those trails of full stops were like balls coming at me that I was batting away. Each ball was a small demon or a negative thought. With each stroke on my typewriter, each distinctive crash on my machine felt like progress. I *was* travelling. I was travelling in my own mind, putting to bed issues and unhelpful comparisons … I began going out again, feeling my feet contact the ground and I was suddenly a part of the environment again. I was lifting my head and looking at the world. Then each night I would go back to my machine for a fresh meditation, albeit a loud one. I was becoming increasingly dextrous on the typewriter and I felt ownership over a skill I had taught myself, over an idea that was mine and mine alone. […] I felt in control again. It felt amazing. I was an artist. I could be a mum and be sane and be an artist and it was all possible from the comfort of my studio (a.k.a my bedroom). It was a revelation, and an empowering one. (Alexander 2018)

Drawing on affect theory (Wasson 2018), the process of making the maps reveals what it feels like to “inhabit” a series of “cryptic present moment[s]” that may “eventually find an as-yet-unknown meaning” (3) – the reference to progress in the retrospective description above suggests some form of narrative coherence for the artist/patient. But before those moments “solidify” into narrative and into a sense of identity (the ‘I’ that recurs above), readers, like Alexander, are confronted with the “strained, equivocal labour” (Ibid, 3–4) of “hammering” a single (ambiguous) key on the typewriter and “batting away the balls.” Through waiting and going back to her “loud meditation,” not unlike Sedgwick’s own meditative practice and “suspended agency” mentioned in this essay’s opening section, Alexander crafts intimate authority, as she becomes increasingly dextrous on her typewriter. Not presented as ennobling or redemptive, this kind of agency is important for communicating Alexander’s personal experience discreetly (and comfortably, as she notes above), bringing it into the public sphere, but also for destigmatizing mental illness: “Those creative choices are mine and are profound to me but as an artist I invite and confront you gently to lean in and take a look … Who is really looking at other people? Was anyone ever looking at me thinking I was an incompetent mother? Are we all inward and all battling our own demons? Certainly. Are we doing ourselves any favours by worrying? Certainly not” (Alexander 2018).

*Mind Maps* shows how an artist can turn maps into “intimate spaces” (Sherwood 2017, 79) that convey emotions and lived experiences through their dotted life-lines. But at the same time Alexander expands established forms of life (and illness) narration. The book format’s sequentiality works well in terms of tracing a life, and in many ways *Mind Maps* embodies the familiar metaphor of ‘life as a journey.’ However, its conceptual approach and accommodation of the episodic through the outline of experiences that lack the larger temporal structure of narrative means that the book communicates powerfully without the need to “appeal to elaborate stories of origins, motives, obstacles and change” (Kirmayer 2000, 155). The fact that “mental health breakdowns” reverts back to zero suggests a kind of closure to the story of Alexander’s post-natal depression (i.e recovery), but *Mind Maps* does not aim to contain a whole life narrative. Ultimately, how the book is read depends on whether readers have the willingness to inhabit those meandering lines of ellipses or full stops, surrendering to their force, movement and pain, without seeking too quickly to join the dots.

## The present being stitched together – over and over

**Fig. 3:**
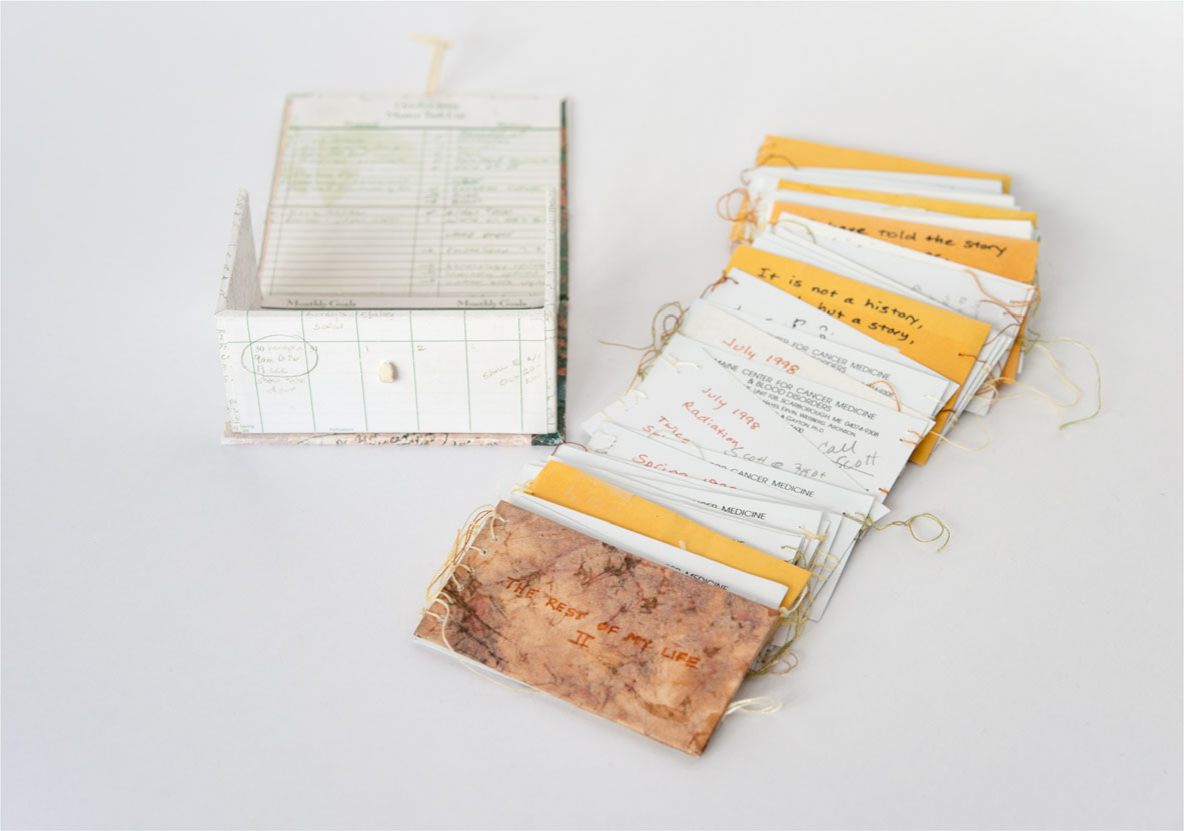
Martha Hall, *The Rest of My Life II* (2003). Handmade box covered in color copies of the artist’s planning calendar, accordion with hand stitching and original paste covers. Maine Women Writers Collection, University of New England, Portland, ME. Photo by Laura Taylor

Rather than simply having a scientific look through its maps and lists, Martha Hall’s *The Rest of My Life II* (2003) is more directly concerned with the world of medicine. Hall created around a hundred artists’ books in response to her initial diagnosis of breast cancer in 1989 and the effects of later recurrences until her death in 2003. A variation of a book she made in 2000, *The Rest of My Life II* is enclosed in a handmade box, making its content unknown until the box is opened, even though the color copies of the artist’s planning calendar that cover the box give readers a glimpse of the book’s preoccupation with time. Once the box is opened, the book can be viewed as a rolodex or taken outside to be examined more closely. The second option reveals a long accordion book bound together with hand stitching. The accordion segments are Hall’s actual appointment cards for 1 year of cancer treatments, October 30, 2000–October 2001. While the dates change, the cards look similar (most are from Maine Center for Cancer Medicine & Blood Disorders) though some of them are different (for example, some refer to dental appointments). Also included are brown manila envelopes that contained medication, which were given to the artist before chemotherapy treatments (one of the envelopes includes the medical label DECADRON, 4 mg tablet and the instruction, “Take 5 tablets 4 hours before chemotherapy”). While not visible, the tablets inside the sealed envelopes can be palpated when touching the envelopes. There are two simultaneous texts in addition to the appointment dates and calendar pages: handwritten notes on everyday events (such as shopping lists and other banal reminders), appearing usually on one side of the brown envelopes and occasionally on the appointment cards themselves; and – what seems to have the highest potential for storytelling compared to the book’s other elements – a history of the past 10 years of the artist’s life with cancer in red pen on the back of the appointment cards. Unlike Alexander’s more restrained codex form, Hall’s book can be opened from either end to read Hall’s account of her life in chronological order or to examine the text on the appointment cards and envelopes (Fig. 3).

Among the series of similar looking appointment cards and envelopes, four hand-written notes (in black pen) on the brown envelopes stand out as readers unfold this book from one end (the one that doesn’t document Hall’s life). Functioning as anchoring points, they not only illuminate aspects of its construction but also the ways in which the book as a whole captures Hall’s lived experience of illness.

Each time I write this history I look

for a pattern.

Repetition.

Repeat.

Pause.

It is not a history,

though, but a story,

a record, a pulse-taking.

the present being stitched

together–over and over.

I have told the story

too many times.

I know its rhythm, but

not its patterns.

The events are threaded

together by tests,

treatment, tests.

I do not know

what comes next.

The first note communicates Hall’s search for pattern. The experience of repeating and pausing matches her medical history as a patient who has to manage cancer as a chronic disease; as we read in one of the notes in red pen from July 1998: “Radiation. Twice a day for two weeks. Skip a week. Repeat 4 times.” But readers of this book encounter another instance of repetition and pause when the appointment cards that constitute this book’s pages are interrupted with a brown envelope. When handling this book, I was tempted to look for a pattern myself, wondering about Hall’s conscious choices as an artist while putting this book together: for example, does an envelope always appear after three cards in a row? While she seems to follow this pattern for a while, she does not adhere to it systematically. And sometimes readers have to pause because one of the cards is arranged in the direction of right to left making it difficult to read the writing on it. If “flow and break is the basic internal dialogue of a book form” (Drucker 2004, 140), Hall self-reflexively draws attention to this feature as she amplifies that tension.

The second note quoted above differentiates between “history” and “story.” Illness stories have emerged as a distinct genre precisely in order to communicate subjective or lived experiences that are not found in medical case histories with their objective and technical style. While Hall includes references to symptoms, tests, and treatments in the “history” part of her book, like Alexander’s outline of varying experiences in *Mind Maps*, she integrates these into a broader life narrative (albeit episodic, as the notes capture selected moments). For example, she refers to personal and professional events such as celebrating her birthday, receiving her MBA from Dartmouth, and working on several of her artists’ books. Even though the appointment cards may be approached as metonyms of the objective or clock time through which her physicians constitute the temporality of cancer as a disease, the book’s “pulse,” to use Hall’s term, testifies to how illness is constituted differently by patients. By representing what seems like an eternal present, notably the repetitive and enduring nature of illness (“This cycle doesn’t end, it just starts over,” as Hall writes in her diary), the book captures in a palpable way the experience of chronic illness: what Hall eloquently describes as “the present being stitched together – over and over.” As in the case of the “parallel chart” (Charon 2006, 155) – though this time written from the patient’s perspective as opposed to how it is originally conceived within narrative medicine – this is an alternative “pulse-taking” that renders medical experience more tangible. Kristin Langellier’s description of the parallel chart in performative rather than narrative terms suits the multimodal nature of artists’ books. The cards in *The Rest of My Life II* “repeat” the medical appointment cards that were given to Hall, the patient, but as “different,” remaking them so that the unfolding space/time of this book becomes a “critical,” and, I would add, aesthetic, “intervention” (Langellier 2009, 155) through which Hall, the artist, claims her own form of intimate authority.

Hall’s third note returns to the idea of rhythm: “I know [the story’s] rhythm, but not its patterns.” The sheer number of medical appointment cards that appear with regularity as readers move through this book communicates how the chronically ill body becomes a regulated body. As Martin O’Brien (2014) writes in a different context, drawing on the concept of “chronic time” by medical sociologist David Morris and on Foucauldian paradigms of bio-power, chronically ill patients with a life-threatening illness not only “endure” their illness but also the “regulation” of their bodies by biomedical regimes to which they voluntarily surrender in order to slow down their “biological demise” (57–8). Hall is aware of this contradiction as one of her diary entries demonstrates. She notes that she chose to recycle these cards for her book because of their intimate size and the fact that they can be “held in hand”; because they show the “passage of time” and highlight “repetition”; and finally, because they are examples of her “accomplishments.” Though it may sound “strange,” as she acknowledges, they are proof that she has “done something,” that “I’ve been a good soldier.” Foucault discusses the soldier as an example of the “self-disciplining,” regulated or “docile body” in *Discipline and Punish*, and of course, illness as a battlefield is a central metaphor in illness narratives even though it has been criticized (Sontag 1991). What allows Hall to claim some kind of control (though not mastery) over the regulation of her body is precisely her *aesthetic* response here, as she constructs *The Rest of my Life II* by recycling her appointment cards. This is a response that is mediated by her illness experience but accumulates further meaning as it “invests” in the rich, imaginative associations of the artist’s book and of book making.

When Hall writes in the fourth note that “the events are threaded together by tests, treatments, tests,” she alludes to a life of management of her disease that involves regular treatments at particular times and surveillance over the body through hospital visits and tests, but also to the structure of the book – literally held together by these threads. It is worth noting here that this kind of connection is more apparent in the case of the first version of *The Rest of My Life* (2000) where the book’s binding is through medical bandage taping rather than stitching. Below is an excerpt from this earlier book that documents the time Hall was diagnosed with a recurrence of breast cancer:

Friends ask, “How much longer?”

They mean, “when will chemo be

done?”

. . .

Now,

what is the answer?

“Never”

“Until I die.”

or

“As long as I live.”

. . .

“I am living with cancer.”

“I am buying time.”

“I have a chronic disease.”

(Hall 2003, 54)

In *The Rest of My Life II*, “buying time” becomes living with “borrowed time.” As Hall explains, the second book is a “tangible reminder” that “there was no scheduled end for this chemotherapy,” no remission or stop to the numerous side effects of treatment but also a “reminder to live fully and in the present.” This shift is also expressed through the new binding of the book. The stitching (done by her daughter Daniella as Hall’s hands by that time were too frail to execute it) is deliberately loose, its ends hanging out, and occasionally tangled, forcing us to slow down and move through the book carefully; it is as if the book, like Hall’s life, hangs on a thread. There are other moments in the text that encourage us to establish a parallel between Hall’s vulnerability and that of the book’s design. For example one of the brown envelopes includes the note (written on the side, in faint ink, as opposed to the clear black ink and central position of the previous notes, making it easier for readers to overlook it): “holding on, coming apart.” Holding on (another way of referring to enduring) and its opposite, whether interpreted as reconciling to the thought of dying or letting go of some ties that belong to the past, is another tension negotiated by *The Rest of My Life II*. The phrase “holding on, coming apart” is therefore a metaphor but also quite literal when readers handle this fragile book.

And yet despite death looming (one of the notes at the back of an appointment card includes funeral instructions: “No urn, woodbox, haystack for ashes, plant elm tree or lupine field…”), as Hall writes in the final note, “I do not know what comes next.” In fact, the book ends with a blank envelope as a way of communicating the unknown future. Hall died in December of the year she created *The Rest of My Life II*, so does the reader fill in this blank? Sequence and finitude are supposed to be two of the main structural features of the book form. In retaining sequential regularity (though not rigid sequence given the two entry points of the book) but refusing closure, *The Rest of My Life II* raises awareness of the complexity of chronic illness experiences. If the more culturally dominant versions of illness narratives “restore to reality its lost coherence and … discover, or create, a meaning that can bind it together again” (Hawkins 1991, 2–3), Hall’s book operates at the critical edges of these assertions. It binds (this time literally) its elements together, but does so loosely. It moves forward, documenting Hall’s history through the clock time of appointment cards that contain different dates, but with its repetitive rhythm, “the stitching of the present –over and over,” it also questions narrative reading, that is, reading “for the direction of its point” (Wasson 2018, 3). Hall may be looking for a pattern, but her book is full of moments that compel her readers to pause and witness the pulse of “an agonised” or “emergent” present (Ibid, 3–4). Hall’s words, “I do not know what comes next,” become then an invitation to stay with those moments and endure their “affective weight” rather than tame them into a narrative of personal meaning, future decline or other telos (Wasson 2018, 5–6).

## The book as meditative practice

While *The Rest of My Life II* communicates the challenging temporality of an ‘eternal present,’ Amanda Watson-Will’s *Like Weather* (2007), an Inkjet-printed limited-edition book that adopts a flag structure, represents and cultivates a different kind of awareness of time. The construction of Hall’s book out of medical appointment cards, as I have shown, captures, and claims aesthetic control over, the chronically ill body’s never-ending regulation by biomedical regimes. *Like Weather* – a book that functions as both document or record of an experience and as conceptual space where a performance can unfold for maker and viewer alike (Watson-Will 2008, 34) – presents us with a similarly disciplined or regular practice of attention to the body. However, from the outset, this practice is not mediated by medicine. In her artist’s statement on her website, Watson-Will describes the act of making as a means to “ground me in the present” and elsewhere explains that the creation of *Like Weather* both reinforced “the practice of mindfulness and facilitated processing of the emotions” she experienced (Watson-Will 2017, 98). Watson-Will came to art in her early thirties after developing chronic fatigue syndrome. Even though *Like Weather* is not about chronic fatigue, it draws on her personal experience to explore “change as a central facet of existence”: “Process, transience, fragility and death can be uncomfortable ideas and yet we must learn to accommodate them in order to live without fear.”

Unlike Hall’s and Alexander’s focus on serious illness, *Like Weather* turns to the more ordinary experience of ‘wrestling’ with thoughts which doesn’t mean that it cannot cause deep anxiety or have implications for one’s wellbeing. While Alexander has described her typewriter art as a form of “loud meditation,” the concept of *Like Weather* is specifically based on the idea from Buddhist meditation that thoughts, feelings, and sensations are constantly changing, drifting across the mind like clouds in a sky. Like weather, which becomes the book’s title, our states of mind are not constant. The book opens with the words, “Buddhists believe …,” written directly on an image of a blue sky with white clouds in the inside front cover of the book and completed by the phrase, “…emotions are like clouds” in the inside back cover. There is both a sense of stillness and movement in this image aided by the fact that the words, arranged as they are on the photo of the sky, give the impression of floating, just like the clouds portrayed in it.

**Fig. 4:**
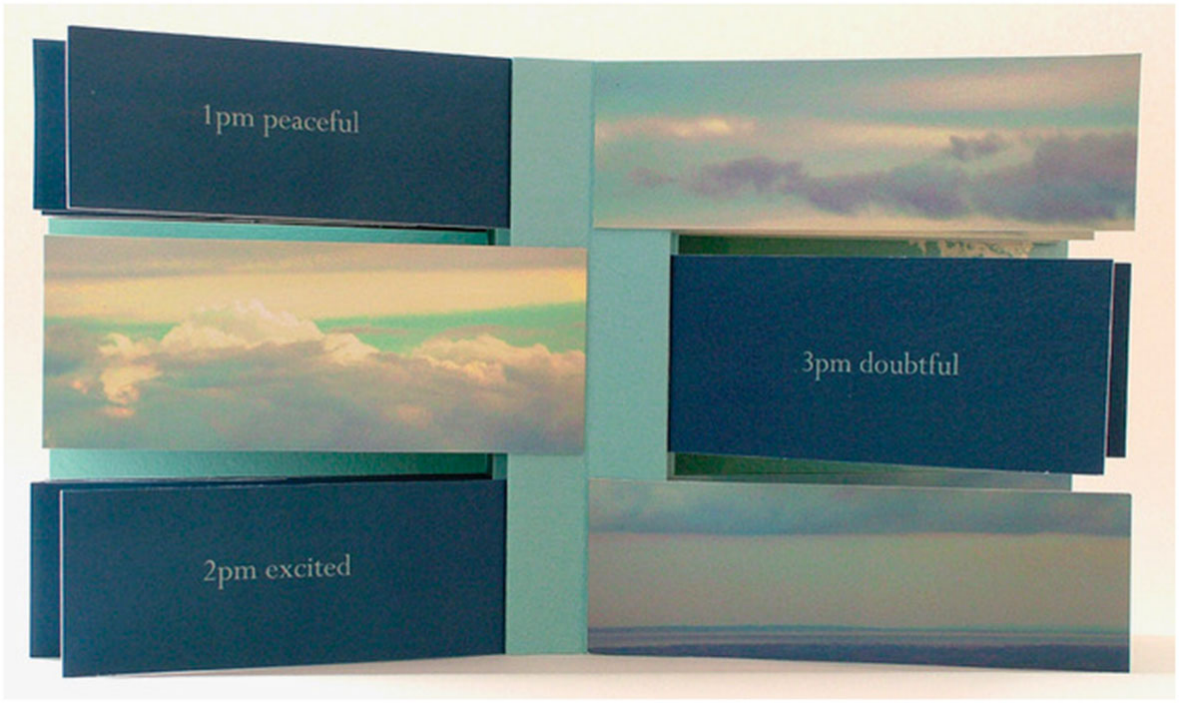
Amanda Watson-Will, *Like Weather* (2007). Archival inkjet prints. Special Collections and Archives, University of Kent. Detail showing image and text in the book’s flags when read page by page

As we have already seen, artists’ books integrate the formal means of their production with their themes, so this framing message is reinforced through the book’s flag structure. *Like Weather*’s flags consist of clouds in the sky, this time presented through repeated crops of the inside cover image, juxtaposed with an hourly record of the artist’s predominant emotion between 11 pm May 14th and 11 pm May 15th, 2007 (for every hour that she was awake). For example: 12 noon down, 1 pm peaceful, 2 pm excited, 3 pm doubtful, 4 pm rushed, 5 pm thoughtful, 6 pm emotional, 7 pm happy and so on. Generally, flag books disrupt unitary sequence, creating a fragmentary effect that results in “complementary or contrasting images and narratives. When read page by page, the viewer sees disjointed fragments of image and text that encourage a moment-focused reading. When the spine is pulled fully open, these fragments assemble a panoramic spread” (Hanmer in Watson-Will 2008, 34). This works well in *Like Weather* to highlight change (cloud-like emotions drifting across the sky) as well as to represent the panoramic sky which is always there irrespective of whether it is momentarily hidden by clouds (Fig. 4).

**Fig. 5:**
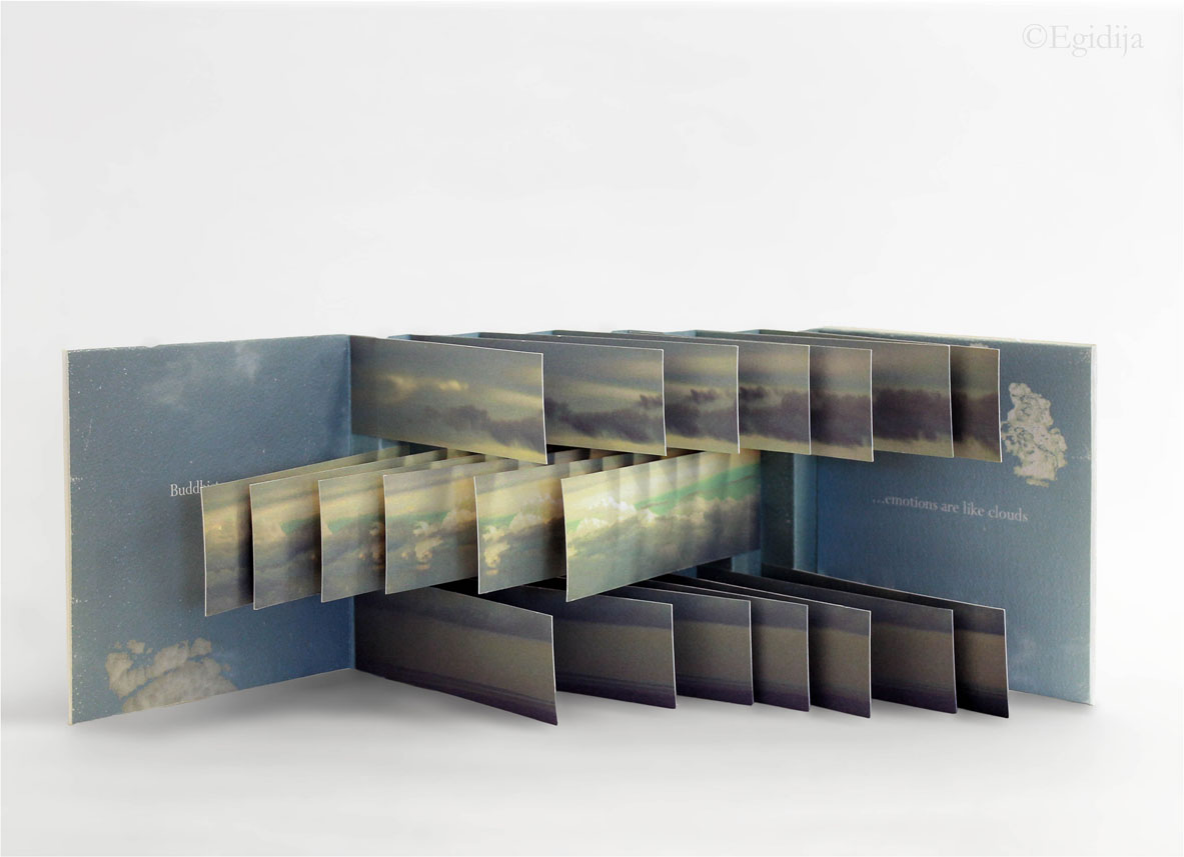
Amanda Watson-Will, *Like Weather* (2007). Archival inkjet prints. Special Collections and Archives, University of Kent. Display of the book with spine pulled fully open. Photo by Egidija Čiricaitė

While giving the book a sculptural dimension, the flag structure “creates an almost imperceptible movement across the image” (Watson-Will 2008, 36) though not in a particular direction as expected of narrative. This movement recalls handling a flip book. In fact, writing in her blog about personal experiences that inspired *Like Weather*, Watson-Will (2009) uses a ‘bookish’ metaphor when she refers to both distressing and happy news she received that “made her ‘*flip’* from a sense of incredible joy and satisfaction to deep desolation” (emphasis added). The book’s flag structure therefore enacts rather than merely providing information about the transient nature of moods and feelings: as readers ‘perform’ this book, they flip from one state to the other. *Like Weather* attests to how creative practices can help make the elusive “phenomenological properties of inner experiences of consciousness” (Sauer et al. 2011, 2) more visible and thus shareable. In this case, the book (especially through its flag structure) can be approached as a realized embodiment of the idea “that prolonged practice of mindfulness may develop the ability to dissect experiences into more subtle parts” (Ibid). Not only does it represent this idea in a concrete way by giving it physical form, but in moving beyond scientific approaches to consciousness, it endows these inner experiences with texture, color, and aesthetic pleasure. All these form part of the intimate authority the artist can claim in having made that book as well as share with others as they read/perform it (Fig. 5).

Watson-Will has made additional artistic choices to refine the book’s exploration of time and subjective mental states while strengthening its links to the spiritual frameworks it draws upon. For example, thought has been put into the choice of images: the variation of clouds in the flags visually complements the fluctuating emotions recorded in the text. At the same time, the “layers of clouds of increasing darkness and the sliver of blue sky peeking through” becomes a metaphor for the hopeful (though not stable) message some readers may extract from the book: “after the winds and rain subside, finer days will be waiting for us” (Watson-Will 2007a). The photographs of clouds are both real and digital to suggest “tension between the real and the virtual, the reality of a situation and our emotional response to it” (Watson-Will 2007b), which meditative practices like mindfulness try to alleviate through an emphasis on observing the change of moods without judging. Printing the images on the page without a border also conveys the interdependence of the self with the sky, a sense of connection and entanglement with the world that many contemplative practices encourage. Like the paper’s texture (Canson 100, a double sided water-color paper coated with Inkaid, an inkjet pre-coat), the imagery with its rich colors but soothing effect adds to the book’s almost mystical experience or wonder and compels our touch. While the work expresses the lack of substance of cloud-like emotions, the slightly more weighty paper contradicts that; the contrast between the theme of ephemerality and the durable, archival chosen materials may surprise us initially. However, considering this book’s link to mindfulness, it is telling of the kind of burden or weight associated with the mind’s natural tendency to over-analyze and over-judge feelings and emotions instead of simply acknowledging their presence or experiencing their weightlessness.

There has been increasing interest in mindfulness within medicine, particularly when it comes to its therapeutic intervention in areas such as mental health as well as in addressing burnout within the medical community. However, there is agreement that “mindfulness has the potential to be more than a medical and psychological tool” for alleviating stress (Lewis 2016, 415). Echoing some of the critiques of the instrumental use of illness narrative, Bradley Lewis writes of the importance of opening up “our medical research to the wider linguistic, religious, and theoretical dimensions of mindfulness” (Ibid, 404). Medical humanities has responded in various ways to these calls to expand limiting approaches – for example, by creating bridges between medical and spiritual approaches to health concerns, often marginalized within Western medicine, via mindfulness (Lewis 2016) or by exploring the link of mindfulness to narrative medicine, reflective practices in medical education, and to compassionate care (Connelly 2005).

Book arts practices can make their own contribution to these ongoing discussions by reclaiming the sensual and creative, if not spiritual, aspects of mindfulness and by furthering a medical/health humanities that “embraces beauty” (Bleakley 2013). Writing in the context of contemporary practices of crafting, with which book arts have affinities, Ann Cvetkovich (2012) suggests that for some people who are skeptical of spiritual matters, it may be easier to think of spiritual practice as “a daily habit or in terms of the more secular category of creative practice” (197). Cvetkovich’s study focuses on depression outside a strictly medical framework, and a large part of the book is about the importance of ordinary embodied “rituals” that do not imply transcendence or escape from the messy realities of life and that can be described in “sensory and affective terms” (Ibid, 197). Crafting is one such ritual, given the forms of repetition it requires. Crafting consists “not of exercising more control over the body and senses but instead of ‘recovering’ them from the mind or integrating them with it. This recovery of the senses infuses life with “sensory pleasure” (Ibid, 168).

Repetition was instrumental to the creation of Alexander’s ‘mind maps’ (she describes her typewriter ritual as a form of meditation, as we have seen), and the same could be said about the construction of Hall’s *The Rest of My Life II*, relying on rhythm as opposed to a recognizable pattern. While not necessarily sharing the forms of repetition typical of crafting or always emerging from the “habitual nature of domestic life” (Cvetkovich 2012, 189), the experience of making artists’ books often occurs in a private and meditative space that encourages full engagement in the present moment. Artists’ books are created for one-on-one interaction, and reading them consists of a *practice*, a multisensory experience of their ‘enchanted’ materiality. This practice often requires modes of attention that resemble those of meditation as, while handling the book readers become slowly aware of the various elements the artist has built into it. This adds “a ritualistic element” to the act of reading and creates “a space for a contemplative experience” (Strand 2017, 90). In the specific context of healthcare, where routine and impersonal interactions frequently turn professionals into automatons, such a space, often silent, where awareness and presence can be restored, is essential.

Even though this gap is being addressed through an emphasis on “reflection,” as Catherine Belling rightly notes, reflection tends to become synonymous with narrative. As she proposes, the “lyric” may be more helpful for cultivating reflection than narrative as it captures the need to “pause the momentum of plot” that is so important to the action-driven field of medicine and to “focus down, observe closely and question deeply” (Belling 2012, 2). Belling does not equate the lyric with poetry and in fact illustrates her argument by reading a novel, that “quintessential narrative form” (Ibid) where the lyric mode can be embedded. Like her, I am suggesting that even though the artist’s book has strong narrative ties and can thus enhance narrative competence, making or reading artists’ books can facilitate an alternative kind of reflection that engages both body and mind (Bolaki 2017, 8). In this way, book arts can redress the dominance of narrative reflective models within healthcare education.

Cautioning against the risk of “reifying” illness narrative by approaching it as a singular “object” or “entity,” Julia E. Connelly (2005) writes that the “practice of mindfulness is a doorway that opens to an awareness of the patient’s story unfolding in the present moment” (84, 92). Considering all three works explored in this essay and the modes of tactile and multisensory engagement they invite, artists’ books powerfully demonstrate that this *unfolding* of experience *in the present moment* – whether agonized, stagnated or grounding – is not merely a metaphor.
